# Role of ADAM and ADAMTS proteases in pathological tissue remodeling

**DOI:** 10.1038/s41420-023-01744-z

**Published:** 2023-12-09

**Authors:** Zhaoni Wang, Wanshan Li, Shixing Chen, Xiao Xiao Tang

**Affiliations:** 1 State Key Laboratory of Respiratory Disease, National Clinical Research Center for Respiratory Disease, National Center for Respiratory Medicine, Guangzhou Institute of Respiratory Health, The First Affiliated Hospital of Guangzhou Medical University, Guangzhou, China; 2 Guangzhou Laboratory, Bio-island, Guangzhou, China

**Keywords:** Pathogenesis, Diseases

## Abstract

Pathological tissue remodeling is closely associated with the occurrence and aggravation of various diseases. A Disintegrin And Metalloproteinases (ADAM), as well as A Disintegrin And Metalloproteinase with ThromboSpondin motifs (ADAMTS), belong to zinc-dependent metalloproteinase superfamily, are involved in a range of pathological states, including cancer metastasis, inflammatory disorders, respiratory diseases and cardiovascular diseases. Mounting studies suggest that ADAM and ADAMTS proteases contribute to the development of tissue remodeling in various diseases, mainly through the regulation of cell proliferation, apoptosis, migration and extracellular matrix remodeling. This review focuses on the roles of ADAM and ADAMTS proteinases in diseases with pathological tissue remodeling, with particular emphasis on the molecular mechanisms through which ADAM and ADAMTS proteins mediate tissue remodeling. Some of these reported proteinases have defined protective or contributing roles in indicated diseases, while their underlying regulation is obscure. Future studies are warranted to better understand the catalytic and non-catalytic functions of ADAM and ADAMTS proteins, as well as to evaluate the efficacy of targeting these proteases in pathological tissue remodeling.

## Facts

- Pathological tissue remodeling is closely associated with the occurrence and aggravation of various diseases.
- ADAM and ADAMTS proteases have contributed to the development of pathological tissue remodeling in many diseases, mainly through the regulation of cell proliferation, apoptosis, migration and extracellular matrix remodeling.
- PI3K/AKT, ERK, EGFR and other signaling pathways are involved in ADAM- and ADAMTS-mediated tissue remodeling.
- Identifying the roles of ADAM and ADAMTS proteases in pathological tissue remodeling may be a promising direction for the treatment of relevant diseases.

## Open questions

- What processes of tissue remodeling are ADAM and ADAMTS proteases involved in?
- What are the molecular mechanisms that ADAM and ADAMTS proteases mediate pathological tissue remodeling?

## Introduction

Generally, tissue remodeling refers to the reorganization or renewal of tissue structures. It could be divided into two categories, one is physiological tissue remodeling, and another is pathological. Physiological tissue remodeling is a normal, programmed process contributing to organ development and functional maturity [1]. Pathological tissue remodeling is defined as a transient process or permanent result of wound healing response. In some clinical diseases, pathological tissue remodeling is considered a sign of refractoriness or poor prognosis, like airway remodeling in severe asthma [2] and usual interstitial pneumonia (UIP) in rheumatoid arthritis [3]. Tissue remodeling could happen in various kinds of tissues or organs, including adipose tissue, airway epithelium, pulmonary interstitium, smooth muscle, and blood vessels. Pathological tissue remodeling presents as, but is not restricted to, overproduction of extracellular matrix (ECM), proliferation of mesenchymal cells (such as smooth muscle cells and myofibroblasts), angiogenesis, etc. [4, 5]. In clinical entities with persistent tissue remodeling, original architecture is broken and replaced with chaotic structure, leading to a deficiency in biological functions. For instance, lung interstitial fibrosis, as a result of an extensive accumulation of (myo)fibroblasts and synthesis of ECM in the pulmonary interstitium, dampens the diffusion of oxygen into alveolar vessels and leads to hypoxia in patients [6].

Zinc-dependent metalloproteinases comprise a superfamily of proteinases, including MMPs (Matrix Metalloproteinases), ADAM (A Disintegrin And Metalloproteinases) and ADAMTS (A Disintegrin And Metalloproteinase with Thrombospondin motifs). Due to the presence of a disintegrin domain, ADAM and ADAMTS constitute the subfamily of adamalysins distinguished from MMP. MMPs and ADAM/ADAMTS share a common function in regulating tissue remodeling through the degradation of matrix or non-matrix proteins. The role of MMPs in tissue remodeling has been well-depicted [7–9], while ADAM and ADAMTS are less discussed and reviewed. Therefore, here we focus on the functions and mechanisms of ADAM and ADAMTS on tissue remodeling in diseases, intending to identify their therapeutic potentials in clinical entities with severe conditions of tissue remodeling.

## Role of ADAM and ADAMTS in pathological tissue remodeling

**Fig. 1:**
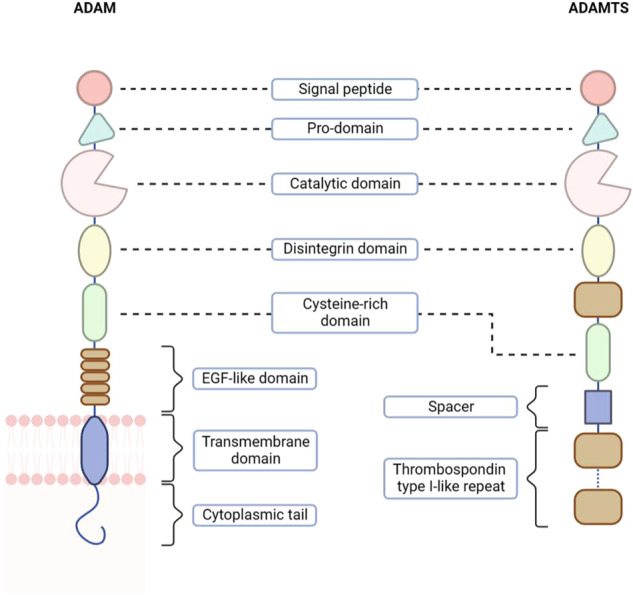
Structural schematics of ADAM and ADAMTS family proteases. ADAM proteins are generally membrane-tethered and consist of eight basic domains, including signal peptides, pro-domain, catalytic domain, disintegrin domain, cysteine-rich domain, EGF-like domain, transmembrane domain and cytoplasmic tail. The common structure of ADAMTS family proteases is largely similar to that of ADAM except for two differences. The first one is the lack of transmembrane domain so that ADAMTS proteins are in secreted form. The second one is the existence of a spacer domain and thrombospondin type-like repeat domain in ADAMTS, and there is a great variability in the number of thrombospondin type-like repeat in the C-terminal between ADAMTS members.

Since the discovery of the first mammalian disintegrin protein (ADAM1) functioned in fertilization in 1987 [10], 21 ADAM family members and 19 ADAMTS proteins have been reported in human during the following years [11, 12]. The common structure of ADAM proteases is composed of a signal peptide, pro-domain, catalytic domain, disintegrin domain, cysteine-rich domain, EGF-like domain, transmembrane domain and cytoplasmic tail. ADAMTS proteins share the same former five domains with ADAM proteases. Differently, they have a thrombospondin type I-like repeat between the disintegrin-like and cysteine-rich domains, followed by a spacer domain and one or more specialist domains, which vary greatly among ADAMTS members [12]. What’s more, unlike ADAM proteases being cell surface-bound, ADAMTS proteins are secreted and detached from the cell membrane due to a lack of transmembrane and cytoplasmic domains (Fig. 1). However, some of the ADAM proteases (such as ADAM28) also present secreted isoforms without transmembrane domain [13].

Generally, zinc-dependent metalloproteinases have the pro-domain that functions in maintaining the latency and directing folding of the enzymes, therefore, activation of proteases requires cleavage of the pro-domain [12, 14, 15]. The catalytic function is the basic function for active ADAM/ADAMTS. However, some of the ADAM proteases have lost this ability due to the absence of a zinc-binding catalytic motif [16]. Based on this proteolytic effect, ADAM/ADAMTS exert their physiological function in many biological processes or diseased conditions. For instance, versican is an ECM component that influences the adhesion, migration and proliferation of many cell types [17]. ADAMTS-mediated versican cleavage is essential for embryogenesis by dismantling the original structure and allowing dynamic remodeling [18]. Nevertheless, increased ADAMTS-specific versican cleavage was also found to be associated with the pathogenesis of disease situations like heart failure [19]. Except for several members that were identified as pseudogenes, functional ADAM metalloproteinases are critical in activating and shedding diverse growth factors, adhesion molecules and matrix proteins through their catalytic activity [12, 16]. Accumulating evidence has demonstrated the contributions of ADAM proteins in pathological conditions such as tissue remodeling [20–23]. In this review, we profile the role and molecular mechanisms of ADAM and ADAMTS proteases in pathological tissue remodeling.

### ADAM/ADMATS and cell proliferation

Extensive proliferation of mesenchymal cells, such as fibroblasts and smooth muscle cells, is considered to be a common process in tissue remodeling. ADAM and ADAMTS proteases have been implicated in oncogenesis by regulating the proliferation of cancer cells [24]. In non-carcinoma diseases, overexpression of ADAM or ADAMTS proteins may regulate abnormal growth and expansion of mesenchymal cells.

The proliferation of vascular smooth muscle cells contributes to the development of cardiovascular and pulmonary diseases. Airway vascular proliferation is one of the key features in airway remodeling. Numerous studies have shown that ADAM33 polymorphism was correlated to the risk of airway diseases, including asthma and chronic obstructive pulmonary disease (COPD) [25–28]. Studies suggested that the proliferative phenotype of airway smooth muscle cells (ASMCs) was maintained by the constitutive expression of ADAM33 [29, 30]. Silencing expression of ADAM33 significantly reduced cell proliferation, possibly via inactivating PI3K/AKT and ERK signaling [29–31]. Pulmonary arterial hypertension (PAH) is characterized by pulmonary vascular remodeling, which is attributed to increased proliferation of pulmonary artery smooth muscle cells (PASMCs). Upregulation of ADAMTS8 was thought to be involved in the pathogenesis of pulmonary vascular remodeling in PAH [32]. Overexpression of ADAMTS8 was mainly discovered in PASMCs of PAH or mouse lungs of hypoxia-induced pulmonary hypertension. Specific deletion of ADAMTS8 in PASMCs ameliorated hypoxia-induced pulmonary hypertension in mice and reduced PASMC proliferation with upregulation of AMP-activated protein kinase (AMPK). Conversely, ADAMTS8 overexpression promoted PASMCs proliferation with a reduction of AMPK. ADAM17 is a multifunctional protease involved in a broad spectrum of diseases including cancer, inflammatory and cardiovascular diseases [33]. ADAM17 contributed to vascular remodeling by elevating the proliferation of vascular smooth muscle cells. Mechanically, this was regulated by phosphorylation of epidermal growth factor receptor (EGFR) at Tyr1068 and ERK1/2 [34, 35]. ADAM17 is also known as tumor necrosis factor-alpha converting enzyme (TACE). IL-13-induced proliferation of human bronchial epithelial cells depended on ADAM17-mediated release of TNF-α [36]. Interestingly, IL-13 did not alter the expression level of ADAM17 but promoted the translocation of TNF-α from intracellular to apical regions of epithelial cells, where ADAM17 was significantly expressed.

### ADAM/ADAMTS and cell apoptosis

Cell apoptosis is involved in the occurrence and development of tissue remodeling. Upon intrinsic or extrinsic damage, apoptosis might be the foremost reaction in suffered cells. When these injuries were persistent, cell apoptosis would also keep happening and lead to disease progression. In the cardiovascular system, chronic Ang II stimulation induces a variety of pathologies, including aneurysmal aortas and PAH. Pathogenically, these two kinds of diseases share the same etiology but have totally different histologic changes. Simply put, one shows vasodilation while another exerts vasoconstriction. Several ADAM and ADAMTS are involved in the underlying mechanisms.

Human aneurysmal aortas present a decrease of ADAM15 and α-SMA. In mouse model, Adam15 deficiency enhanced Ang II-induced structural deterioration in the aortic wall, accompanied by increased apoptosis of aortic smooth muscle cells (ASMCs). However, Ang II promoted the proliferation of wild-type ASMCs rather than apoptosis. These findings indicated that ADAM15 deficiency was essential in this phenotype conversion and directed the disease development. Under Ang II stimulation, the deletion of Adam15 facilitated the expression of thrombospondin 1 (THBS1), which could activate the cofilin pathway, increase G-actin, and induce apoptosis of ASMCs [21]. In another study, a decrease of ADAMTS5 was found in acute aortic dissection (AAD), which was associated with apoptosis of ASMCs. In vitro experiments showed that recombinant ADAMTS5 protected ASMCs from Ang II-induced cell apoptosis, while the underlying mechanism was unclear [37, 38]. In diabetic cardiomyopathy, decreased myocardial apoptosis by conditional deletion of Adam17 ameliorates myocardial remodeling and dysfunction. ADAM17 may function in the cleavage of angiotensin-converting enzyme 2 (ACE2) and inactivation of AMPK, thus decreasing autophagy formation, blocking autophagy flux and finally enhancing apoptosis of cardiomyocytes [20].

### ADAM/ADMATS and cell migration

Cell migration is also an important player during the process of pathological tissue remodeling. Migration and accumulation of structural cells like smooth muscle cells and fibroblasts can lead to thickening or hypertrophy of local tissues. Migration of immune cells to the injured areas results in inflammatory infiltration and may accelerate tissue remodeling.

Upregulation of ADAM33 was implicated in airway vascular remodeling in asthma. In human ASMCs, ADAM33 silencing significantly reduced cell migration, probably via inhibiting the activation of PI3K/AKT signaling, phosphorylation of mammalian target of rapamycin (mTOR), Rho-associated protein kinases, phospho-forkhead box protein O1 (FOXO1), and cyclin D1 [39]. Deficiency of Adam17 suppressed Ang II-mediated migration of VSMCs, possibly through inhibition of EGFR and Erk1/2 signaling [35]. Upon vascular injury, ADAMTS7 was upregulated and mainly located in VSMCs. Migration of primary VSMCs was significantly accelerated by ADAMTS7 overexpression but suppressed by small interfering RNA knockdown [40]. ADAMTS7-mediated cell migration was closely associated with the degradation of vascular ECM cartilage oligomeric matrix protein (COMP), evidenced by that COMP supplementation could avoid the pro-migration effect of ADAMTS7 on VSMCs [40].

Migration of fibroblasts contributes to tissue fibrosis. Shedding of ephrin-B2 was required for fibroblast migration and formation of lung/skin fibrosis through activation of EphB3 and/or EphB4 receptor signaling. A study found that ADAM10 is a major sheddase of ephrin-B2, and targeting ADAM10 with its inhibitor GI254023X could attenuate bleomycin-induced lung fibrosis in mice [41]. ADAM12 has been implicated in the activation of growth factors, such as heparin-binding epidermal growth factor-like growth factor (HB-EGF) and insulin growth factor (IGF) binding proteins. ADAM12 was elevated in chronic skin wounds, accompanied by a reduced migration rate of keratinocytes. However, the absence of ADAM12 markedly accelerated keratinocyte migration and wound healing [42]. ADAM12 also could be induced by an allergen challenge. In airway epithelial cells (AECs) from patients with allergic rhinitis, TNF-alpha increased the expression of ADAM12 in vitro, and this effect could be enhanced by Th2 cytokines, including IL-4 and IL-13. Overexpression of ADAM12 promoted the release of chemokines like CXCL1 and CXCL8 in AECs, and reduced expression of CD47 in neutrophils, which regulated neutrophil adhesion, thus led to migration and recruitment of neutrophils. These effects were mainly dependent on EGFR activation [43]. ADAM8 was also associated with increased migration of immune cells. Deletion of ADAM8 in T cells significantly ameliorated asthmatic response. This was due to the impaired migration of immune cells, including T cells and eosinophils, from blood vessels to the airway and alveolar space, indicating a general hematopoietic cell deficiency in the absence of ADAM8 [44].

### ADAM/ADAMTS and matrix remodeling

Cleavage, disarray, reduction and deposition of extracellular matrix proteins are important steps in aberrant wound healing and result in chaotic tissue structures. Matrix proteins like aggrecan and versican are common substrates for ADAMTS [45]. Intracranial aneurysm (IA) is a common cerebrovascular disease characterized by ECM degradation and remodeling in vessel walls. ADAMTS5 was found to be downregulated in human subjects and mouse model of IA. Inhibition of ADAMTS5 in a zebrafish model led to intracranial hemorrhage in embryos. However, administration of ADAMTS5 recombinant protein attenuated proteoglycan accumulation and elastic fiber destruction in artery tissue of IA. These findings suggested that the protective role of ADAMTS5 in IA was mainly due to its regulation of matrix degradation and matrix remodeling [46]. Transient upregulation of ADAM15 in the myocardium plays a crucial role in reparative post-infarction scarring. As compared to wildtype mice, Adam15-knockout mice showed disordered and decreased fibrillar collagen density, associated with lower levels of insoluble collagen, lysyl oxidase-1 (LOX-1) and fibronectin after myocardial infarction. These effects may be achieved through the downregulation of p21-activated kinase 1 (PAK1), which regulates the expression of fibronectin and LOX-1 [47]. A recent study identified several novel ADAMTS1/4/5 cleavage sites in versican [48]. ADAMTS1 can cleave the large glycoprotein versican at multiple sites in cultured human aortic VSMCs, which may promote atherogenesis [49]. Loss of catalytic function in ADAMTS5 directed the formation of aortic dilatation under Ang II stimulation. Proteomic analysis revealed versican as the most upregulated matrix protein in mice lacking the catalytic domain of ADAMTS5, indicating that versican accumulation in vessel walls promoted the formation of aortic aneurysm, and ADAMTS5 may prevent the development of aortic aneurysm by degradation of versican [50].

## Link between substrate cleavage and biological processes in tissue remodeling

**Fig. 2:**
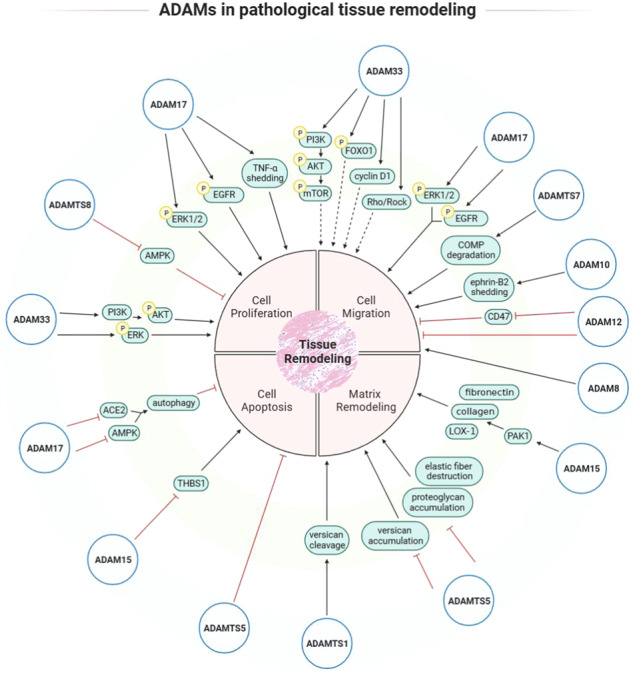
Contributions and mechanisms of ADAM and ADAMTS proteases in pathological tissue remodeling. In a variety of diseases with aberrant tissue remodeling, ADAM and ADAMTS proteins play protective or pathologic roles by influencing biological behaviors (proliferation, migration and apoptosis) of mesenchymal cells or immune cells and reconstruction of extracellular matrix. These effects may be mediated by activation/inhibition of cellular signaling pathways and catalytic degradation of extracellular matrix.

In this review, we describe that catalytic activities of ADAM10 and ADAMTS7, like shedding of ephrin-B2 by ADAM10 and degradation of COMP by ADAMTS7, have been determined to be contributors in cell migration during tissue remodeling (Fig. 2). However, among the literature reporting other ADAM/ADMATS, few have shown the causal role of substrate cleavage on the biological processes correlated to tissue remodeling, including cell proliferation, apoptosis and migration. In order to explore whether the proteolytic effect of ADAM/ADAMTS involves tissue remodeling, we present some studies that may hint at the connection between ADAM/ADAMTS-induced substrate cleavage or shedding and the processes involved in tissue remodeling.

Zou et al. examined the catalytic capacity of human ADAM33 and identified four substrates, including insulin B, stem cell factor (SCF, c-kit), TNF-related activation-induced cytokine (TRANCE) and β-amyloid precursor protein (APP) [51]. Among these substrates, SCF/c-kit plays a critical role in cell proliferation and its expression is increased in PAH [52]. However, whether and how ADAM33-induced SCF/c-kit cleavage gets involved in tissue remodeling is still unknown and requires further investigation. Similarly, the substrate of ADAMTS8, Osteopontin, was also upregulated in PAH and involved in the proliferation of cardiac cells as well as VSMCs [53–55]. This evidence points to a possibility that ADAMTS8 may control the proliferation of mesenchymal cells (e.g., smooth muscle cells) in PAH through osteopontin cleavage.

ADAM15, ADAM17 and ADAMTS5, as we discussed in this review, exert protective roles in cell apoptosis (Fig. 2). Cleavage of some substrates may account for this function. For example, ADAM15 protects ASMCs from Ang II-induced apoptosis [21]. E-cadherin, which can be cleaved by ADAM15 [56], is able to bind specifically to death receptors and regulate cell apoptosis in cancer. And the loss of E-cadherin attenuated apoptosis signaling via the death receptors [57]. Based on these studies, it is highly possible that ADAM15 may prevent cell apoptosis by cleaving the cell-surface protein E-cadherin.

ADAM and ADAMTS have been related to cancer development and metastasis [24]. Since tumor diseases share common biological processes, including cell proliferation, migration and apoptosis with tissue remodeling, investigations of carcinoma may provide valuable insights into the relationship between ADAM/ADAMTS and tissue remodeling. For instance, according to the mechanisms of tumor cell migration and invasion, ADAM proteinases may direct cell migration through the shedding of cell adhesion molecules or modifying of cell-matrix interaction. Potential substrates involved in cell migration have been summarized in previous reviews [58, 59]. Similarly, molecules like ADAM17 have a great number of substrates and are involved in diverse diseases and pathological processes. Some reviews have introduced in detail their potential functions on different biological processes and underlying proteolytic mechanisms [60–62]. The literature is of high reference value for future studies on connections between ADAM/ADAMTS-mediated substrate cleavage and processes related to tissue remodeling.

## Summary

In this review, we summarize the contributions of ADAM and ADAMTS proteases to aberrant tissue remodeling. By regulating the processes of cell proliferation, cell migration, cell apoptosis and extracellular matrix remodeling, ADAM and ADAMTS play protective or inductive roles in the pathogenesis of diseases with tissue remodeling (Fig. 2). Although several members of the ADAM and ADAMTS families have been identified as candidate therapeutic targets for tissue remodeling, the underlying mechanisms of their regulation have not been fully elucidated. The major function of ADAM and ADAMTS lies in their catalytic activities, while their substrates of different ADAM/ADAMTS under different pathological conditions need to be defined in future studies. Of note, non-catalytic domains should also be focused on since some ADAM/ADAMTS can mediate cell adhesion or ADAM-substrate interaction. The growing amount of genomic and proteomic data may provide us an opportunity to better understand the molecular mechanisms of ADAM-mediated tissue remodeling, as well as to explore therapeutic target regions on the ADAM molecules.
